# Attenuation of teratoma formation by p27 overexpression in induced pluripotent stem cells

**DOI:** 10.1186/s13287-016-0286-3

**Published:** 2016-02-15

**Authors:** Toru Matsu-ura, Hiroshi Sasaki, Motoi Okada, Katsuhiko Mikoshiba, Muhammad Ashraf

**Affiliations:** Department of Pathology and Laboratory Medicine, University of Cincinnati, Cincinnati, OH 45267-0529 USA; Laboratory for Developmental Neurobiology, RIKEN Brain Science Institute, Saitama, 351-0198 Japan; Department of Pharmacology, University of Illinois at Chicago College of Medicine, 835 South Wolcott Ave, Chicago, IL 60612 USA

**Keywords:** Stem cell, Induced pluripotent stem cell, iPS, Embryonic stem cell, ES, Cell cycle, cdk, p27, Tumor, Infarcted heart

## Abstract

**Background:**

Pluripotent stem cells, such as embryonic stem cells or induced pluripotent stem cells, have a great potential for regenerative medicine. Induced pluripotent stem cells, in particular, are suitable for replacement of tissue by autologous transplantation. However, tumorigenicity is a major risk in clinical application of both embryonic stem cells and induced pluripotent stem cells. This study explores the possibility of manipulating the cell cycle for inhibition of tumorigenicity.

**Methods:**

We genetically modified mouse induced pluripotent stem cells (miPSCs) to overexpress p27 tumor suppressor and examined their proliferation rate, gene expression, cardiac differentiation, tumorigenicity, and therapeutic potential in a mouse model of coronary artery ligation.

**Results:**

Overexpression of p27 inhibited cell division of miPSCs, and that inhibition was dependent on the expression level of p27. p27 overexpressing miPSCs had pluripotency characteristics but lost stemness earlier than normal miPSCs during embryoid body and teratoma formation. These cellular characteristics led to none or smaller teratoma when the cells were injected into nude mice. Transplantation of both miPSCs and p27 overexpressing miPSCs into the infarcted mouse heart reduced the infarction size and improved left ventricular function.

**Conclusions:**

The overexpression of p27 attenuated tumorigenicity by reducing proliferation and earlier loss of stemness of miPSCs. The overexpression of p27 did not affect pluripotency and differentiation characteristics of miPSC. Therefore, regulation of the proliferation rate of miPSCs offers great therapeutic potential for repair of the injured myocardium.

**Electronic supplementary material:**

The online version of this article (doi:10.1186/s13287-016-0286-3) contains supplementary material, which is available to authorized users.

## Background

Cell therapy with embryonic stem cells (ESCs) or induced pluripotent stem cells (iPSCs) is one of the promising cures to repair damaged tissues and organs in a variety of clinical settings [1]. However, the major concern for the clinical use of ESCs and iPSCs is their ability to induce teratoma derived from all three germ layers when injected into immunodeficient mice [2]. Teratoma formation is caused by the self-renewal capability of undifferentiated transplanted cells. Therefore, predifferentiation of the stem cells and elimination of undifferentiated stem cells before transplantation are important considerations to prevent potential tumorigenesis [3, 4]. However, it is rather difficult to remove the relatively few undifferentiated cells completely from millions of differentiating cells.

One of the main causes of tumorigenesis is uncontrolled cell cycle progression. Cell cycle progression is governed by cyclin dependent kinases (cdk) that are activated by cyclin binding and inhibited by the cdk inhibitors [5, 6]. p27 is one of the major members of the cdk inhibitor protein family and inhibits the cyclin–CDK complex; for example, cyclin E-cdk2, and cyclin D-cdk4 and -cdk6 complexes [7–9]. Studies with *p27* knockout mice that developed multiple organ hyperplasia indicate that p27 has antiproliferative activity [10]. It is reported that reduced expression of p27 is frequently observed in various cancers [11–13] and correlates with poor prognosis [14–16]. From these studies, it is clear that p27 is a promising target for tumor therapies. In fact, it is reported that overexpression of p27 suppresses tumor growth in various cancers [13, 17, 18] and reduces proliferation of human and murine ESCs [19, 20].

In this report, we overexpressed p27 in mouse iPSCs (miPSCs) and explored its potential for reducing the proliferation rate of miPSCs in vitro and in vivo and their differentiation into cardiac phenotypes. Furthermore, the therapeutic potential for reducing myocardial infarction in vivo was also investigated.

## Methods

### Animals

All animal experiments conformed to The Guidelines for Care and Use of Laboratory Animals published by the US National Institutes of Health (NIH Publication No. 85–23, revised 1985), and all protocols of animal experiments were approved by the Institutional Animal Care and Use Committee, University of Cincinnati.

### Materials

The lentivirus vector for expression of mVenus-hGemnin (pCSII-EF-mVenus-hGem) was provided by Dr. Atsushi Miyawaki (RIKEN, BSI) [21]. Monomeric red fluorescent protein (mRFP) [22] gene cloned into pcDNA3 (Invitrogen) was a gift from Dr. Doug Golenbock (University of Massachusetts Medical School) (Addgene Plasmid 13032). Mouse ESCs (mESCs) from C57BL/6 mice were purchased from GIBCO (S1503-100).

### Gene construction

CMV-promoter and neomycin resistance genes were cloned into the SalI site of pSMPUW (CellBiolab) to make pSMPUWneo. *EF1α* promoter was cloned from pEF-E2-Crimson (Clontech) and inserted into XhoI-EcoRI site of pTetOff (Clontech) to make pTetOff-EF1α. The *EF1α* promoter and tet-responsible transcriptional activator (*tTA*) from pTetOff-EF1α were cloned into SalI site of pSMPUWneo to make pSMPUWneo-TetOff. *p27* gene was cloned from rat cDNA. p27 and mRFP were connected with *Thoseaasigna* virus 2A peptide and inserted in ERI-BamHI site of pTRET-tight (Clontech). The *TRET* promoter and *p27-2A-mRFP* were cut out from pTRE-tight and inserted in pGEM-T-easy (Promega). The *TRET* promoter and *p27-2A-mRFP* were cut out from pGEM-T-easy and inserted into EcoRI site of pSMPUWneo-TetOff to make pSMPUWneo-TRET-p27-TetOff.

### Transient and stable transfection

To produce lentivirus, pCSII-EF-mVenus-hGem, pCgpV (Cell Biolabs), pRsv-Rev (Cell Biolabs), and pCMV-VSVG (Cell Biolabs) were transfected to HEK293Ta cells. The supernatant was concentrated using an ultracentrifuge and added to mESCs. mVenus-hGemini expressing clone was selected using fluorescence. To establish the stable cell line of p27-2A-mRFP, pSMPUWneo-TRET-p27-TetOff was digested by XhoI, and the linearized plasmid was transfected into miPSCs [23] with Lipofectamine 2000 (Life Technologies). p27-2A-mRFP expressing clone was selected by resistance to Geneticin (100 μg/ml; Roche) at first, then selection was done using fluorescent active cell sorting (FACS). The stable cell line was designated as miPSCs-p27. Transient transfection of pSMPUW-TRET-p27-TetOff to miPSCs and mESCs was done with Xfect (Clontech), Lipofectamine 2000 (Life Technologies), or Lipofectamine 3000 (Life Technologies) transfection reagents.

### Cell culture, cell proliferation, trypan blue staining, embryoid body formation, and spontaneous cardiac differentiation

miPSCs from C57BL/6 mice [23] and mESCs were cultured with knockout Dulbecco’s modified Eagle’s medium (DMEM; Life Technologies) supplemented with leukemia inhibitory factor (LIF; Millipore), 15 % knockout serum replacement, nonessential amino acids, L-glutamine, 2-mercaptoethanol, and penicillin/streptomycin (Life Technologies). To determine the proliferation rate, miPSCs and miPSCs-p27 were dispersed by accutase (Life Technologies). Cells were then stained with trypan blue (Life Technologies) and counted manually using a hematocytometer. Trypan blue stained cells were counted as dead cells.

Embryoid body (EB) formation was performed as described [24]. Briefly, miPSCs were dispersed into single cells with accutase and were suspended with differentiation medium which consisted of high-glucose DMEM (Fisher Scientific) supplemented with 20 % fetal bovine serum (Life Technologies), nonessential amino acids, L-glutamine, 2-mercaptoethanol, and penicillin/streptomycin. The cells were then transferred to plastic Petri dishes, where they aggregated to form EBs and were cultured in suspension for 9 days.

Spontaneous differentiation from miPSCs to cardiomyocytes was performed as described [25]. Briefly, 500 miPSCs or 1000 miPSCs-p27 in 20 μl aliquots suspended in differentiation medium were cultured in hanging drops to form EBs for 2 days. Individual EBs were transferred to a 96-well ultralow attachment plate (Corning) and incubated for 3 days. EBs were then transferred to gelatin-coated 48-well culture plates and incubated for 9 days. To evaluate the differentiation efficiency of miPSCs and miPSCs-p27, 48-well plates were monitored each day under a microscope to detect the appearance of spontaneously contracting cardiomyocytes.

### Immunocytochemistry

p27-2A-mRFP transient expressing miPSCs were fixed by 4 % formaldehyde and stained by DAPI for detecting mRFP expression and nuclei. To detect overexpression of p27, fixed p27-2A-mRFP transient expressing miPSCs were incubated with rabbit anti-p27 antibody (Abcam, ab32034) and stained by Alexa Fluor 488 conjugate goat anti-rabbit antibody (Life Technologies) and DAPI.

### Imaging

Image acquisition was performed with an inverted microscope (IX71 Olympus, BioStation Nikon). For time-lapse imaging, miPSCs (4 × 10^4^) were seeded on 35-mm glass bottom dishes covered with mouse embryonic fibroblasts (MEFs). After 6 hours of incubation, pSMPUW-TRET-P27-TetOff was transfected to the cells. On the next day, the dish was examined on a BioStation (Nikon), and real time fluorescent imaging was performed to count the divisions of individual mRFP expressing cells with 5 % CO_2_ at 37 °C for 24 hours. Data analysis was performed with ImageJ for IX71 and Nis-Element (Nikon) for Biostation.

### RNA isolation and reverse transcription

Total RNA was purified using an RNeasy kit (Qiagen); 0.2 μg of total RNA was used for the reverse transcription reaction with Omniscript RT kit (Qiagen) according to the manufacturer’s instructions. Polymerase chain reaction (PCR) was performed with Taq DNA polymerase (Qiagen). The primer sequences used are given in Additional file 1: Table S1.

### Flow cytometry analysis

miPSCs or miPSCs-p27 (1 × 10^6^ cells) were seeded onto MEFs in a 60-mm dish. After 2 days, the cells were dissociated using Accutase (Life Technologies) and fixed in 80 % ethanol for 1 hour. Fixed cells were washed with phosphate-buffered saline (PBS) and then resuspended with DAPI-containing solution (1 μg/ml DAPI, 0.1 % Triton X-100 in PBS) for 30 minutes at room temperature for nuclear staining. The stained cells were analyzed by FACSAria II (BD). More than 10,000 cells were collected for analysis of each cell cycle phase. The number of each cell cycle phase was analyzed by the Dean and Jett model [26] with Igor pro software (WaveMetrics).

### Teratoma formation

Immunodeficient nude mice were purchased from Jackson Laboratories, USA. The procedure for assessing teratoma formation in nude mice was approved by the University of Cincinnati Animal Care And Use Committee. Undifferentiated miPSCs or miPSCs-p27 (1 × 10^6^ cells) were suspended with 100 μl of matrigel (BD) and were injected subcutaneously into 2- to 3-month-old nude mice. Four weeks after injection, teratomas were fixed with 10 % formalin and processed for hematoxylin and eosin staining for histological evaluation.

### Myocardial infarction and cell transplantation

A model of acute myocardial infarction was developed in allogenic 8- to 12-week-old male C57BL/6 J immunocompetent mice as described earlier [27]. Briefly, the animals were anesthetized (ketamine/xylazine 80 mg/kg and 20 mg/kg body weight, respectively, intraperitoneally), intubated, and mechanically ventilated (Harvard Rodent Ventilator, Model 683). Minimally invasive thoracotomy was performed for permanent ligation of the left anterior descending artery (LAD) with a Prolene #8.0 suture. Myocardial ischemia was confirmed by color change of the left ventricular wall. The animals were grouped as follows: group 1 was untreated (control, n = 5); intramyocardial injection of 10 μl basal DMEM containing 1 × 10^5^ cells from EBs derived from miPSCs or miPSCs-p27 were performed for the animals of group 2 (n = 4) or group 3 (n = 5), respectively. The cells were injected 10 minutes after LAD ligation at the peri-infarction site in the free wall of the left ventricle (LV) under direct vision. The chest was closed and the animals were allowed to recover. Subsequently, the animals were injected with buprinex (0.05 ml subcutaneously) during the first 24 hours to alleviate pain and were maintained until 4 weeks before euthanasia and sampling of the heart tissue.

### Physiologic assessment of heart function

Transthoracic echocardiography was performed to evaluate heart function at 4 weeks after the respective treatments using iE33 equipped with a 15- to 7-MHz broadband transducer (Philips Ultrasound, Bothell, WA). Intraperitoneal anesthesia was administered with 0.1 % ketamine and 0.02 % xylene per body weight (g) for anesthesia. The animals (group1, n = 5; group 2, n = 4; group 3, n = 5) were anesthetized (approximately 3 minutes) and placed supine onto an imaging platform. The distal extremities were taped gently to electro pads that provide continuous electrocardiographic and heart rate (pulse detection) measurements. The heart was imaged in the two-dimensional mode in the parasternal long-axis and/or parasternal short-axis views, which were subsequently used to position the M-mode cursor perpendicular to the ventricular septum and LV posterior wall, after which M-mode images were obtained. For each animal, measurements were obtained from 4–5 consecutive heart cycles. Measurements of ventricular anterior thickness, LV internal dimension, and LV posterior wall thickness were made from two-dimensionally directed M-mode images of the LV in both systole and diastole. Ejection fraction was measured based on the area–length method after measurement of LV internal area and distance from apex to basal LV in both systole and diastole. The scoring system was patterned after the American Society of Echocardiography’s scoring system which is used conventionally in interpreting clinical echocardiographic studies.

### Histological studies

For measurement of infarction size (and area of fibrosis), the hearts were arrested in diastole by intravenous injection of cadmium chloride and fixed in 10 % buffered formalin. The heart was excised, cut transversely, and embedded in paraffin for Masson’s trichrome staining. Infarct size was defined as the sum of the epicardial and endocardial infarct circumference divided by sum of the total LV epicardial and endocardial circumferences using computer-based planimetry with Image-J analysis software (version 1.6; NIH). The pathology specimens of teratomas were assessed by a pathologist.

### Statistical analysis

All values are expressed as mean ± standard error. Statistical difference between the considered groups was evaluated by Student’s *t*-test or Tukey-Kramer multiple-comparison test. A *P* value <0.05 was considered significant.

## Results

### Inhibition of proliferation in p27 expressing miPSCs

**Fig. 1:**
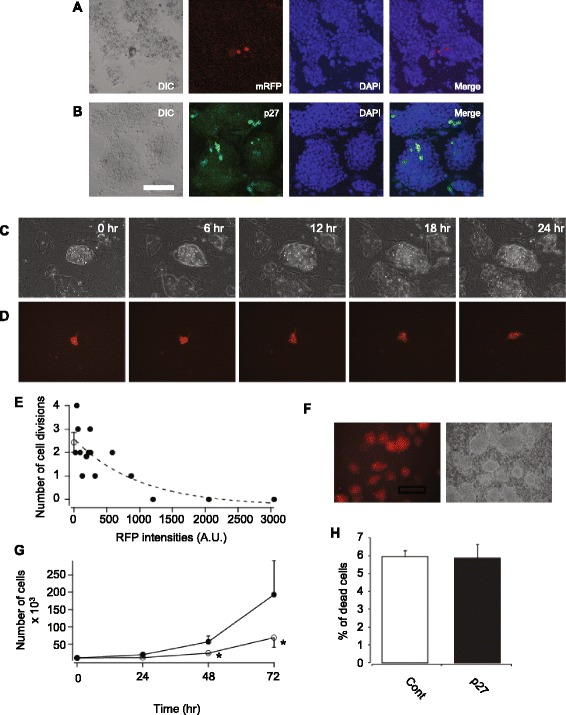
Proliferation of p27 overexpressing miPSCs. Overexpression of p27-2A-mRFP in transient transfected miPSCs are shown with mRFP fluorescence (**a**) and immunostaining of p27 (**b**) with nuclear staining by DAPI. The bar represents 100 μm. **c**, **d** Time lapse images of phase contrast (**c**) and mRFP (**d**) in p27-2A-mRFP transfected miPSCs. **e** Relationship between the number of cell divisions and intensities of mRFP in p27-2A-mRFP expressing miPSCs (*closed circles*). An *open circle* shows the average of the number of cell divisions in control miPSCs (n = 3). *Dotted line* shows fitting line of results of p27-2A-mRFP expressing cells with exponential function. **f** Phase contrast (*left*) and mRFP fluorescent (*right*) images of miPSCs-p27. **g** Proliferation rate of miPSCs (*closed circles*) and miPSCs-p27 (*open circles*). **h** Comparison of the percentages of trypan blue stained cells between miPSCs and miPSCs-p27. Error bars correspond to the SEM (n = 3). *P < 0.05, student’s *t*-test. *mRFP* monomeric red fluorescent protein

cDNA of *p27* in miPSCs was transiently transfected to investigate the effect of p27 overexpression. p27 expressing cells could be detected with red fluorescence by combining cDNAs of *p27* and *mRFP* with *2Apeptide* gene. The overexpressions of mRFP and p27 were confirmed by fluorescence of mRFP and immunostaining of p27 (Fig. 1a and b). Figure 1c and d shows time lapse images of proliferation of the transient transfected miPSCs over 24 hours. Because of the low transfection efficiency (~1 %), most of the miPSCs did not exhibit mRFP expression. Phase contrast images show expansion of the surface area of miPSC colonies due to proliferation of cells (Fig. 1c). On the contrary, the p27 overexpressing miPSCs did not divide throughout the imaging period (Fig. 1d). Figure 1e shows the relationship between fluorescent intensity of mRFP (expression level of p27) and number of cell divisions over 24 hours with a negative correlation. In the mRFP intensity range of 0 to 500, some cells showed a higher number of divisions than those of control miPSCs (2.4 ± 0.4). In the mRFP intensity range of 500 to 3000, all cells showed a lower number of divisions than those in control miPSCs. The p27 overexpressing cells with mRFP intensities more than 1000 exhibited no cell division during real time imaging (Fig. 1e). To maximize the number of miPSCs overexpressing p27, a stable p27 overexpressing cell line was developed (miPSCs-p27; Fig. 1f) using FACS (Figure S1 in Additional file 2). The proliferation rate of miPSCs-p27 was slower than that of miPSCs. The number of miPSCs-p27 cells 3 days after seeding on gelatin coated dishes was less than that of miPSCs (Fig. 1g). Because the percentage of trypan blue stained miPSCs-p27 was the same as that of miPSCs, the reduced proliferation rate in miPSCs-p27 was not due to cell death (Fig. 1h).

### The population of G1 phase cells was increased in miPSCs-p27

**Fig. 2:**
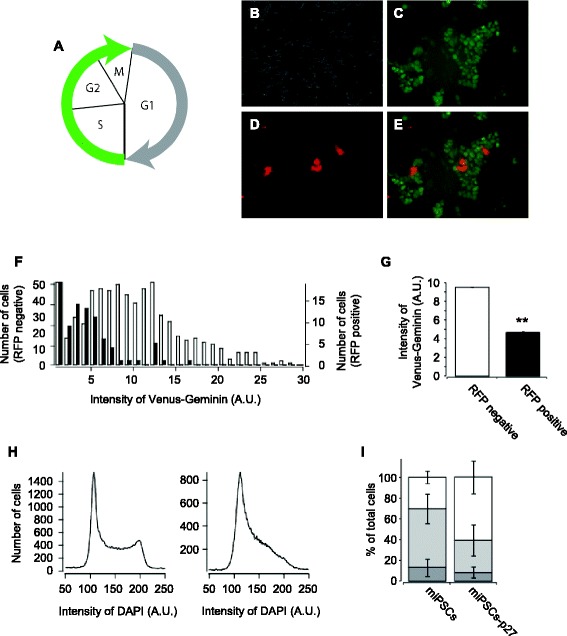
The cell cycle of p27 overexpressing mESCs. **a** Schematic representation of the cell cycle sensor, Fucci. Expression of mVenus-hGeminin can be seen during S to M phases. **b**–**e** Images of phase contrast (**b**), mVenus (**c**), mRFP (**d**), and merge of mVenus and mRFP (**e**) of p27-2A-mRFP transfected mESCs stably expressing mVenus-hGeminin. **f** Profiles of fluorescent intensities of mVenus-hGeminin in p27-2A-mRFP expressing (*black bars*) and nonexpressing (*white bars*) mESCs. **g** Averaged fluorescent intensities of mVenus-hGeminin in p27-2A-mRFP expressing and nonexpressing mESCs. Error bars correspond to the SEM (n = 3). **P < 0.01, student’s *t*-test. **h** Flow cytometry data of DAPI stained miPSCs (*left*) and miPSCs-p27 (*right*). **i** Populations of each phase of cell cycle in miPSCs and miPSCs-p27. *White bars* show G1, *gray bars* show S, and *dark gray bars* show G2 phases. Error bars correspond to the SEM (n = 3). *miPSC* mouse induced pluripotent stem cell, *miPSCs-p27* p27 overexpressing mouse induced pluripotent stem cells, *RFP* red fluorescent protein

Since the proliferation rate of miPSCs was decreased by p27 overexpression, we investigated the effect of cell cycle phases in pluripotent stem cells by p27 overexpression. First, we used mESCs to assess the effect of p27 overexpression on cell cycle progression in pluripotent stem cells. To measure the cell cycle changes, we made a cell line which stably expressed a cell cycle sensor mVenus-hGeminin in mESCs [21]. Expression level of hGeminin oscillates during proliferation of the cells due to cell cycle-dependent proteolysis by ubuiquitin-proteasomal systems. hGeminin level is high during S to M phases and low in G1 phase (Fig. 2a) [21]. The degree of hGeminin expression was not uniform amongst the cells (Fig. 2b and c) because cell cycles of mESCs were not synchronized. We observed that most of the p27 expressing red fluorescent cells had low green fluorescence (Fig. 2d and e). Figure 2f and g shows profiles and average values of mVenus fluorescence, respectively. Both of the graphs clearly show a low level of green fluorescence amongst the population of p27 overexpressing mESCs compared to p27 nonoverexpressing cells (Fig. 2f and g). This result suggests that p27 overexpression resulted in more cells at G1 phase in mESCs.

miPSCs-p27 also showed an increase in the population in the G1 phase by p27 overexpression. Figure 2h shows the flow cytometry analysis of miPSCs-p27. There were two peaks in the flow cytometry results of miPSCs in the left panel of Fig. 2h. The left and right peaks originated from the cells in G1 and G2 phases, respectively. The cells between the peaks represented the cells in S phase. Compared to miPSCs, the G1 peak was wider in miPSCs-p27 (right panel in Fig. 2h). Figure 2i shows the number of cells in each phase of the cell cycle. There were significant differences in G1 and S phases between miPSCs-p27 and miPSCs (G1: miPSCs 30.2 ± 6.1 %, miPSCs-p27 60.8 ± 15.8 %, *P* < 0.01; S: miPSCs 56.5 ± 14.1 %, miPSCs-p27 30.8 ± 15.2 %, *P* < 0.05; student *t*-test). These results clearly show that overexpression of p27 arrested more cells at G1 phase, which was consistent with the ability of p27 to suppress G1–S transition by inhibition of cyclin D and E [28].

### EB formation and cardiac differentiation of miPSCs-p27

**Fig. 3:**
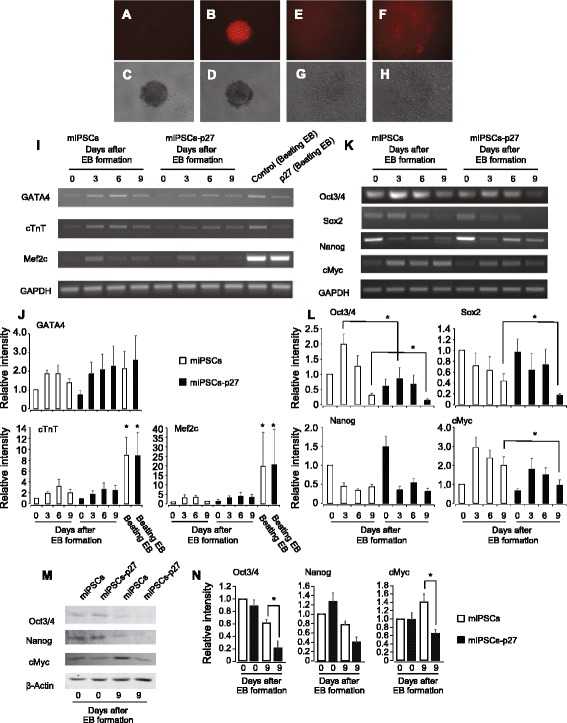
Differentiation of miPSCs-p27. **a**–**d** Images of EBs from miPSCs and miPSCs-p27 on the sixth day of EB formation. Fluorescent images of miPSCs and miPSCs-p27 are shown in (**a**) and (**b**), respectively. Phase contrast images of miPSCs and miPSCs-p27 are shown in (**c**) and (**d**), respectively. **e**–**h** Images of beating EBs from miPSCs and miPSCs-p27. Fluorescent images of miPSCs and miPSCs-p27 are shown in (**e**) and (**f**), respectively. Phase contrast images of miPSCs and miPSCs-p27 are shown in (**g**) and (**h**), respectively. **i**, **j** Expression of GATA-4, cTnT, and Mef2c after EB formation is shown. RT-PCR analysis of the genes (**i**) and relative intensities of the mRNA to GAPDH expression (**j**) of EBs from miPSCs and miPSCs-p27 are shown. High expressions of the genes were detected in beating EBs derived from miPSCs or miPSCs-p27. *P < 0.05, comparison to 0 days of miPSCs with Tukey-Kramer test. **k**, **l** Expression of pluripotent marker genes are shown. RT-PCR analysis of stem cell marker genes (**k**) and relative intensities (**l**) of miPSCs and miPSCs-p27 are shown. *P < 0.05, student’s *t*-test. Error bars correspond to the SEM (n = 3). **m**, **n** Protein expression of pluripotent marker genes are shown. Western blots of stem cell marker genes (**m**) and relative intensities (**n**) of miPSCs and miPSCs-p27 are shown. *P < 0.05, student’s *t*-test. Error bars correspond to the SEM (n = 3). *EB* embryoid body, *miPSC* mouse induced pluripotent stem cell, *miPSCs-p27* p27 overexpressing mouse induced pluripotent stem cells

RT-PCR analysis of pluripotent marker genes was performed to investigate the effect of p27 overexpression on pluripotency of miPSCs. Expression of *p27* in miPSCs-p27 was almost 10 times higher than that of miPSCs (Figure S2A in Additional file 3) and results were consistent with the results of p27 overexpression in mESCs and p27 null miPSCs [19, 29]. There was no significant difference in expression of *Oct4*, *Nanog*, *Sox2*, and *cMyc* in miPSCs and miPSCs-p27 (Figure S2A and B in Additional file 3), suggesting that p27 overexpression did not affect the pluripotency characteristics of miPSCs. This was determined in miPSCs and miPSCs-p27 to test the effect of p27 overexpression on differentiation. To account for low proliferation, a two-fold higher initial cell number was used for miPSCs-p27 compared to miPSCs in hanging drops. miPSCs-p27 formed EBs with a shape similar to miPSCs (Fig. 3a–d). The limited number of EBs formed by miPSCs-p27 might be due to their slower proliferation rate (Figure S3A in Additional file 4). Spontaneously beating EBs were observed both from miPSCs and miPSCs-p27 (Fig. 3f–h). There was no difference in the frequency of spontaneously beating EBs amongst miPSCs and miPSCs-p27 (Figure S3B in Additional file 4), suggesting no inherent difference in their ability to differentiate into cardiac myocytes. Linearized plasmid DNA tends to integrate into the transcriptional active site of host genome. Therefore, the expression of transgene is sometimes lost after differentiation. We observed a distinct mRFP signal in EBs arising from miPSCs-p27 (Fig. 3b). However, the expression of mRFP was not clear in differentiated cardiac myocytes (Fig. 3f), suggesting that the transgene was integrated into a stem cell-specific gene locus. The induction of cardiac marker genes (*GATA4*, *cTnT*, *Mef2c*) in miPSCs-p27 were similar to EBs from miPSCs as determined by EB formation assays and spontaneous cardiac differentiation assays (Fig. 3i and j). The expression of pluripotency genes using EB formation assay was different in miPSCs and miPSCs-p27. The expression of *Oct4*, *Sox2*, and *Nanog* was reduced during EB formation. There was a significant decrease in *Oct4* and *Sox2* on the ninth day of EB formation by p27 overexpressing iPSCs (Fig. 3k and l). Compared to the initial expression level, *cMyc* expression was three times higher on the third day of EB formation by miPSCs. In contrast, the induction of *cMyc* expression was 1.5 times higher on the third day in miPSCs-p27. The expression of *cMyc* was gradually decreased both in miPSCs and miPSCs-p27, and it was significantly less in miPSCs-p27 than miPSCs on the ninth day of EB formation (Fig. 3k and l). Similar results were also obtained at the protein level for Oct4, Nanog, and cMyc on the ninth day of EB formation by p27 overexpressing iPSCs (Fig. 3m and n).

### Teratoma formation by miPSCs-p27

**Fig. 4:**
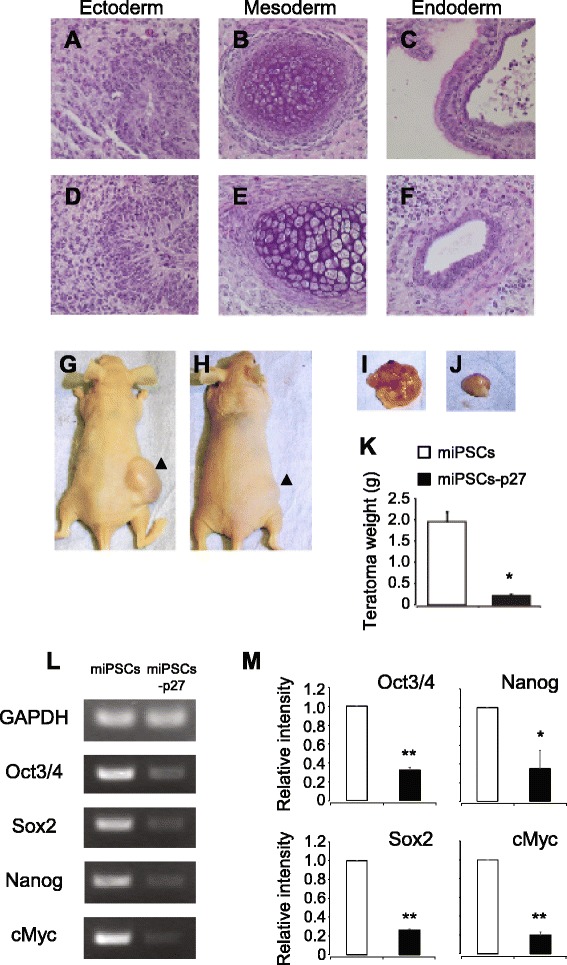
Teratoma formation of p27 expressing pluripotent stem cells. **a**–**f** Representative images of sections from miPSCs or miPSCs-p27 derived teratomas. Hematoxylin and eosin staining of teratoma derived from miPSCs (**a**–**c**) and miPSCs-P27 (**d**–**f**). Teratomas contained tissues derived from all three germ layers. **g**, **h** Teratomas were formed by subcutaneous injection of miPSCs (**g**) and miPSCs-p27 (**h**) into nude mice. **i, j** Teratomas from miPSCs (**i**) and miPSCs-p27 (**j**) injected nude mice. **k** Average weight of teratomas from miPSC and miPSC-p27 injected nude mice. **l**, **m** Expression of pluripotent marker genes are shown. RT-PCR analysis of stem cell marker genes (**l**) and relative intensities (**m**) of miPSCs and miPSCs-p27 are shown. Error bars correspond to the SEM (n = 3). *P < 0.05, student’s *t*-test. *miPSC* mouse induced pluripotent stem cell, *miPSCs-p27* p27 overexpressing mouse induced pluripotent stem cells

miPSCs-p27 normally differentiated into cardiac myocytes despite the presence of pluripotency genes. Teratomas were formed in mice injected with either miPSCs or miPSCs-p27, and comprised of tissues consisting of three germ layers (Fig. 4a–f), consistent with RT-PCR data. The sizes of teratomas in miPSCs-p27 injected nude mice were significantly smaller than that of miPSC injected nude mice (Fig. 4g–k). Four and seven independent experiments were done for miPSCs and miPSCs-p27, respectively. However, while we observed teratomas in all miPSC injected mice, we could find teratomas only in four of seven miPSCs-p27 injected mice (see Figure S4 in Additional file 5 for all teratomas from miPSCs or miPSCs-p27). Transient overexpression of p27 by intramuscular injection of miPSCs also attenuated the teratoma formation (Figure S5 in Additional file 6). Figure 4l and m shows the expression of pluripotent marker genes in teratomas of miPSC and miPSC-p27 injected nude mice. Teratomas contained undifferentiated stem cells as detected by expression of *Oct4*, *Sox2*, *Nanog*, and *cMyc*. However, the expression levels of pluripotency genes were significantly less in miPSC-p27 derived teratomas (Fig. 4l and m).

### Effect of miPSCs-p27 on functional recovery of the ischemic hearts in vivo

**Fig. 5:**
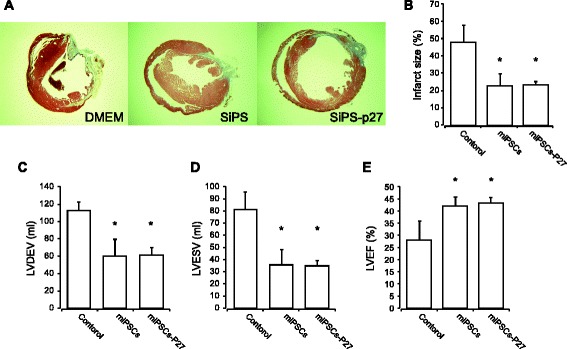
Effect of miPSCs-p27 on functional recovery of ischemic heart in vivo*.* **a**, **b** Histologic assessment of LV cross section after Masson’s trichrome staining which stained visible scar region as blue. Infarction size was significantly reduced in miPSC and miPSC-p27 injection groups as compared with the DMEM injected group showing extensive scar formation. *P < 0.05. **c**–**e** Functional recovery of LV was significantly improved by miPSC and miPSC-p27 transplantation, whereas DMEM transplantation groups showed loss of function as analyzed by (**c**) LV end-diastolic volume (*LVDEV*), **d** LV end-systolic volume (*LVESV*), and (**e**) LV ejection fraction (*LVEF*) using echocardiography. *P < 0.05. *miPSC* mouse induced pluripotent stem cell, *miPSCs-p27* p27 overexpressing mouse induced pluripotent stem cells

To evaluate the therapeutic potential of miPSCs-p27 for the recovery of cardiac function in the ischemic hearts in vivo, we transplanted miPSCs or miPSCs-p27 into the infarcted mouse hearts. All animals survived the full length of the study, and there were no deaths related to cell transplantation. Animals (DMEM, n = 5; miPSCs, n = 5; and miPSCs-p27, n = 4) were euthanized at 4 weeks after the respective treatments. Transplantation of both miPSCs and miPSCs-p27 in and around the infarcted myocardium attenuated infarct size expansion compared with the DMEM-treated group (DMEM, 47.9 ± 9.9 %; miPSCs, 22.9 ± 7.0 %; and miPSCs-p27, 23.3 ± 2.1 %; Fig. 5a and b). We also performed echocardiography to evaluate LV function after cell transplantation. The indexes of LV contractile function were well preserved in the miPSC and miPSC-p27 groups, including LV end-diastolic volume (DMEM, 112.2 ± 5.7 ml; miPSCs, 60.1 ± 9.8 ml; and miPSCs-p27, 61.3 ± 4.1 ml; Fig. 5c), LV end-systolic volume (DMEM, 81.2 ± 6.7 ml; miPSCs, 35.1 ± 6.4 ml; and miPSCs-p27, 34.7 ± 2.2 ml; Fig. 5d), and LV ejection fraction (DMEM, 28.0 ± 3.6 %; miPSCs, 40.7 ± 2.3 %; and miPSCs-p27, 43.2 ± 1.2 %; Fig. 5e).

## Discussion

Transplantation of pluripotent stem cells in the infarcted heart produces malignant tumors. The tumorigenicity is a major risk of cell therapy and is due to abnormal proliferation of pluripotent stem cells. Therefore, prevention of proliferation is a potentially effective approach for clinical use of pluripotent stem cells. Accordingly, we proposed that the overexpression of p27 in miPSCs would reduce tumorigenicity. We show that overexpression of the cdk inhibitor p27 reduced proliferation of mouse pluripotent stem cells and increased the population of the cells in the cell cycle at the G1 phase. The overexpression of p27 slowed transition from G1 to S phase and increased the population of the cells in the G1 phase. These results are consistent with studies on p27 overexpression in cancer cells, and also human and mouse ESCs [17, 19, 20]. It is known that the protein level of p27 is largely regulated by proteolysis [30] which rises during G1 phase progression while the protein level is kept low through the S/G/M phases. The proteolysis may affect the p27 protein level in miPSCs-p27 and promotes slower cell cycle progression.

p27 overexpression did not affect composition of pluripotency genes of miPSCs-p27. Our results are consistent with other reports that overexpression of p27 did not affect the expression of pluripotency genes in human ESCs [19] and is also not sufficient to exit from the pluripotent state in mESCs [20]. miPSCs from p27 knockout mouse showed the same levels of Sox2 and Nanog expression as that of wild-type miPSCs [29]. Furthermore, we found p27 overexpression did not affect the differentiation of miPSCs to cardiac myocytes. These results suggest that overexpression of p27 is a novel experimental approach for suppression of tumorigenicity by pluripotent stem cells without affecting pluripotency. It has been reported that the EBs from skeletal muscle cell-derived miPSCs attenuated infarct size and improved cardiac function when injected into infarcted hearts [23], and our results support that miPSCs-p27 were equally effective. Thus miPSCs-p27 after enhancing expression of cardiac genes with pharmacological or hypoxic preconditioning may become potential candidates for myogenesis in the infarcted hearts with limited chances of tumor formation. Pluripotent stem cells are not only useful to replenish dying cardiac myocytes but also to protect cardiac myocytes from apoptosis via secretion of paracrine factors [31, 32]. Hence, it is also more likely that miPSCs-p27 secrete paracrine factors to protect cardiac myocytes during the critical period of cell death after infarction.

A close relationship exists between cell cycle and differentiation in stem cells. The cdk inhibition promotes early differentiation in stem cells [25–28]. Furthermore, artificial cell cycle arrest by the addition of a CDK-2 inhibitor, roscovitine [33], or genotoxic reagents [34] induces rapid differentiation of stem cells. Therefore, the overexpression of p27 makes them able to induce differentiation by inhibition of the cell cycle progression. In fact, we found faster disappearance of pluripotency genes in EBs and teratomas by p27 overexpression. Also, the number of undifferentiated cells in miPSCs-p27 derived teratomas was reduced. These results are consistent with previous studies showing differentiation in human intestinal epithelial cells and Xenopus primary neurons by p27 overexpression [35]. Similarly, antiproliferation activity and induction of differentiation must be the key functions of p27 to attenuate teratoma formation in miPSCs. miPSCs-p27 sometimes made smaller teratomas, suggesting that p27 is not a perfect solution to prevent teratoma formation. miPSCs-p27 is a heterogeneous cell line in the context of genomic integration sites of the transgene. Hence, expression level and timing of inactivation during differentiation may vary in each cell, thus increasing the chances of teratoma formation. Specific genomic locus targeted gene integration technologies like clustered regularly interspaced short palindromic repeats (CRISPR)-CRISPR associated proteins (CRISPR-Cas9) [36], transcription activator-like effector nucleases (TALEN) [37], and zinc finger nucleases (ZFN) [38] may further improve the genetic modifications in pluripotent stem cells.

Contrary to the early reduction of pluripotency genes in miPSCs-p27 during differentiation, we could not observe any differences in expression of pluripotency genes in miPSCs and miPSCs-p27 cultured on MEFs. The upregulation of the pluripotency genes could be caused by LIF which is secreted from MEFs [39]. The roles of LIF in miPSCs-p27 and cell cycle inhibition in the differentiation of pluripotent stem cells need further investigation.

## Conclusions

We conclude that p27 overexpression leads to a reduced proliferation rate and early loss of stemness during differentiation of miPSCs. Transplantation of EBs developed from both miPSCs-p27 and miPSCs reduces the infarction size following LAD ligation but with smaller-sized teratoma by miPSCs-P27. Further modification of miPSCs-p27 as discussed may render these cells as viable therapeutic options for cell therapy of myocardial infarction and other diseases.

## Additional files

Additional file 1: Table S1. Specific primer sequences for RT-PCR analyses. (DOCX 12 kb) Additional file 2: Figure S1. Purification of p27 expressing miPSCs. Flow cytometric analysis of miPSCs (A), or p27-2A-mRFP stable transfected miPSCs (miPSCs-p27) before (B) and after (C) purification by cell sorter. Intensities of mRFP fluorescent were plotted against side scatters (SSC). The area surrounded by red line in B was sorted. (PDF 348 kb) Additional file 3: Figure S2. Effects of p27 overexpression on EB formation. Percentages of wells which have EBs and beating EBs are shown in A and B, respectively. Error bars correspond to the SEM (n = 3). **P* < 0.05, student’s *t*-test. (PDF 341 kb) Additional file 4: Figure S3. Expression profile of stem cell factors in miPSCs-p27. RT-PCR analysis of stem cell marker genes (A) and relative intensities (B) of miPSCs and miPSCs-p27 are shown. Error bars correspond to the SEM (n = 3). **P* < 0.05, student’s *t*-test. (PDF 311 kb) Additional file 5: Figure S4. All teratomas from miPSCs or miPSCs-p27 injected in nude mice. Teratomas from miPSCs and miPSCs-p27 injected mice are shown (A–D) and (E–H), respectively. (PDF 828 kb) Additional file 6: Figure S5. Teratoma formations of miPSCs or p27 transient transfected miPSCs injected in nude mice. Phase contrast image (A) and fluorescent image (B) of miPSCs transfected with p27-2A-mRFP. Teratomas were shown 4 weeks after transplantation at right hind limb. miPSCs (C) or p27-2A-mRFP (D) transfected miPSCs were intramuscular injected into nude mice. (PDF 460 kb)

## Abbreviations

cdk: Cyclin dependent kinase
DMEM: Dulbecco’s modified Eagle’s medium
EB: Embryoid body
ESC: Embryonic stem cell
FACS: Fluorescent active cell sorting
iPSC: Induced pluripotent stem cell
LAD: Left anterior descending artery
LIF: Leukemia inhibitory factor
LV: Left ventricle/ventricular
MEF: Mouse embryonic fibroblast
mESC: Mouse embryonic stem cell
miPSC: Mouse induced pluripotent stem cell
miPSCs-p27: p27 overexpressing mouse induced pluripotent stem cells
mRFP: Monomeric red fluorescent protein
PBS: Phosphate-buffered saline
PCR: Polymerase chain reaction
