# Supplementary material for: Attenuation of teratoma formation by p27 overexpression in induced pluripotent stem cells
Source: Stem Cell Res Ther. 2016 Feb 15;7:30. doi: 10.1186/s13287-016-0286-3 (PMC4754927; doi:10.1186/s13287-016-0286-3)
Supplement: Additional file 1: Table S1. — Specific primer sequences for RT-PCR analyses. (DOCX 12 kb) [file 13287_2016_286_MOESM1_ESM.docx]

**Table S1**. Specific primer sequences for RT-PCR analyses

| Target gene | RT-PCR primer sequences^a^ (5’ to 3’) | Product size (bp) |
| --- | --- | --- |
| Oct4 | F CACGAGTGAAAGCAACTCA | 290 |
|  | R AGATGGTGGTCTGGCTGAAC |  |
| Sox2 | F CACCAACTCGGAGATCAGCAA | 190 |
|  | R CTCCGGGAAGCGTGTACTTA |  |
| Naonog | F GCACCAACTCAACTTCTGAGC | 286 |
|  | R CTCGAGAGTAGCCACCATATC |  |
| cMyc | F GCCCAGTGAGGATATCTGGA | 226 |
|  | R ATCGCAGATGAAGCTCTGGT |  |
| GATA4 | F CTCGATATGTTTGATGACTTCT | 345 |
|  | R CGTTTTCTGGTTTGAATCCC |  |
| cTnT | F GCGAAGAGTGGGAAGAGACA | 127 |
|  | R CCACACAGCTCCTTGGCCTTCT |  |
| Mef2c | F GTATGTCTCCTGGTGTAACA | 370 |
|  | R GGATATCCTCCCATTCCTTG |  |
| GAPDH | F TGGCCTTCCGTGTTCCTACC | 300 |
|  | R TGTAGGCCATGAGGTCCACCAC |  |
| p27 | F ATGTCAAACGTGAGAGTGTCTAACG | 591 |
|  | R CGTCTGGCGTCGAAGGCCGG |  |

F and R denote foreword and reverse primers, respectively.
