# Supplementary figures and images for: Attenuation of teratoma formation by p27 overexpression in induced pluripotent stem cells
Source: Stem Cell Res Ther. 2016 Feb 15;7:30. doi: 10.1186/s13287-016-0286-3 (PMC4754927; doi:10.1186/s13287-016-0286-3)

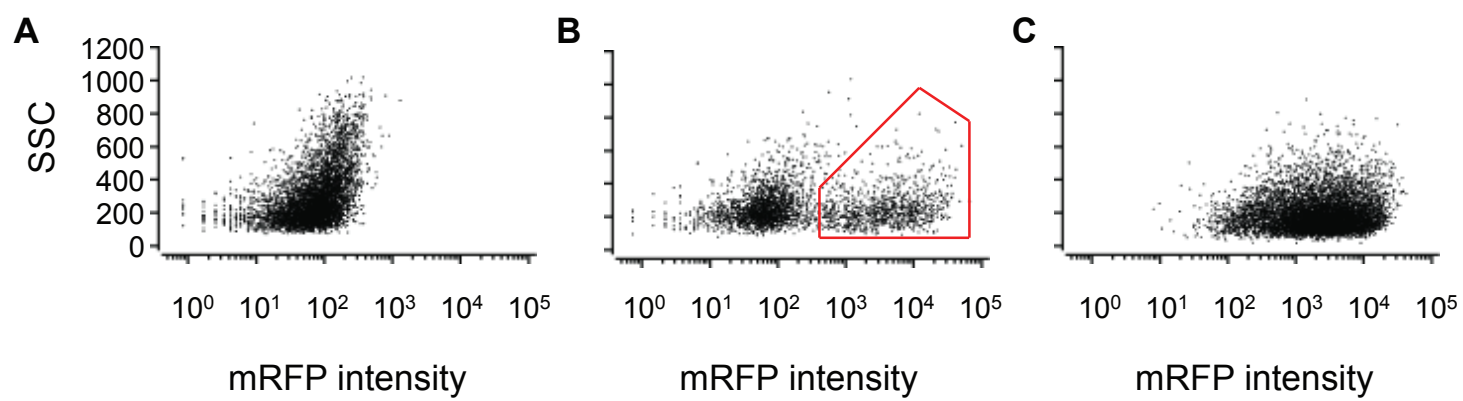

Figure S1

Supplement: Additional file 2: Figure S1. — Purification of p27 expressing miPSCs. Flow cytometric analysis of miPSCs (A), or p27-2A-mRFP stable transfected miPSCs (miPSCs-p27) before (B) and after (C) purification by cell sorter. Intensities of mRFP fluorescent were plotted against side scatters (SSC). The area surrounded by red line in B was sorted. (PDF 348 kb) [file 13287_2016_286_MOESM2_ESM.pdf]

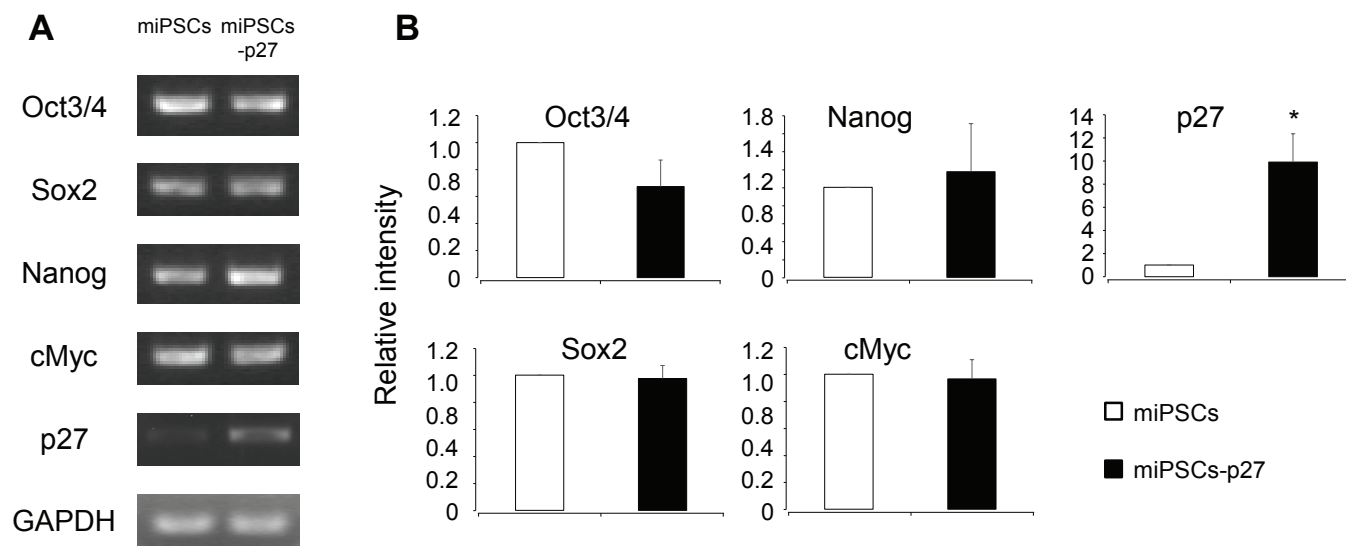

Figure S2

Supplement: Additional file 3: Figure S2. — Effects of p27 overexpression on EB formation. Percentages of wells which have EBs and beating EBs are shown in A and B, respectively. Error bars correspond to the SEM (n = 3). *P < 0.05, student’s t-test. (PDF 341 kb) [file 13287_2016_286_MOESM3_ESM.pdf]

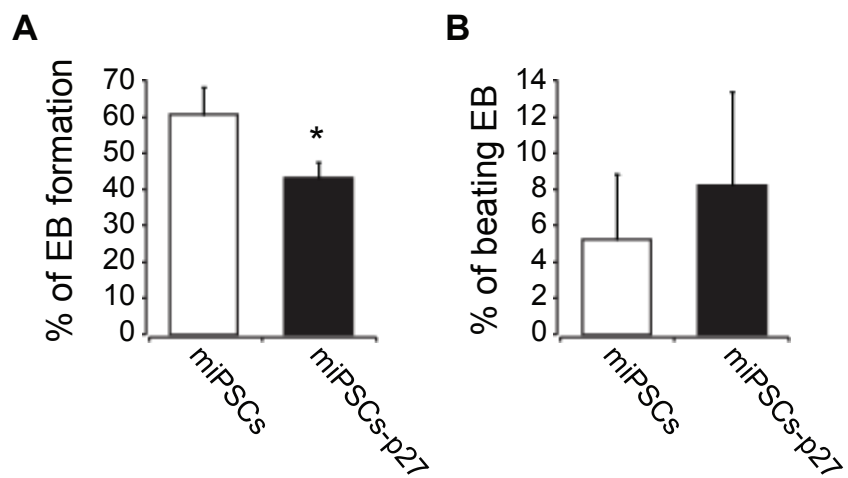

Figure S3

Supplement: Additional file 4: Figure S3. — Expression profile of stem cell factors in miPSCs-p27. RT-PCR analysis of stem cell marker genes (A) and relative intensities (B) of miPSCs and miPSCs-p27 are shown. Error bars correspond to the SEM (n = 3). *P < 0.05, student’s t-test. (PDF 311 kb) [file 13287_2016_286_MOESM4_ESM.pdf]

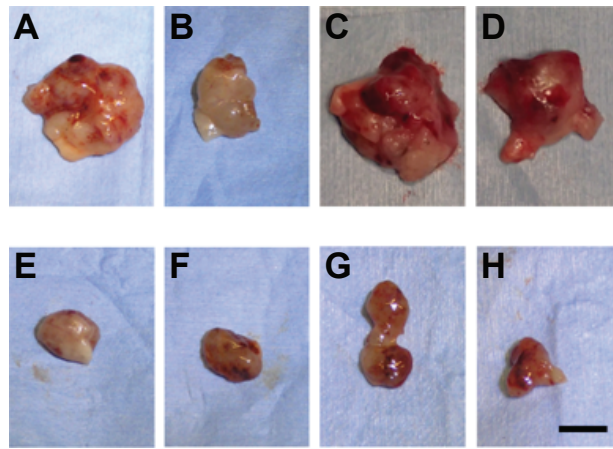

Figure S4

Supplement: Additional file 5: Figure S4. — All teratomas from miPSCs or miPSCs-p27 injected in nude mice. Teratomas from miPSCs and miPSCs-p27 injected mice are shown (A–D) and (E–H), respectively. (PDF 828 kb) [file 13287_2016_286_MOESM5_ESM.pdf]

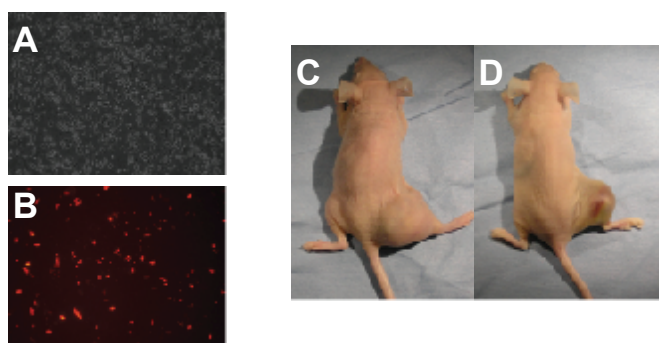

Figure S5

Supplement: Additional file 6: Figure S5. — Teratoma formations of miPSCs or p27 transient transfected miPSCs injected in nude mice. Phase contrast image (A) and fluorescent image (B) of miPSCs transfected with p27-2A-mRFP. Teratomas were shown 4 weeks after transplantation at right hind limb. miPSCs (C) or p27-2A-mRFP (D) transfected miPSCs were intramuscular injected into nude mice. (PDF 460 kb) [file 13287_2016_286_MOESM6_ESM.pdf]
